# Towards a More Holistic Comparative Assessment of Plant-Based Alternative Beverages and Dairy Milk: A True Cost Accounting Approach

**DOI:** 10.3390/foods14132196

**Published:** 2025-06-23

**Authors:** Mauricio R. Bellon, Nicholas Benard, Jane E. Coghlan, Kathleen Merrigan

**Affiliations:** Swette Center for Sustainable Food Systems, Arizona State University, 777 East University Dr, Tempe, AZ 85281, USAkathleen.merrigan@asu.edu (K.M.)

**Keywords:** plant-based alternative beverages, dairy milk, global warming potential, dietary risks, forced labor, monetization, true cost accounting

## Abstract

There is a growing market for plant-based alternative beverages (PBAs) promoted as alternatives to dairy milk. Part of their popularity is that consumers consider them better for both the environment and human health. These perceptions, however, may not be entirely supported by scientific evidence. A holistic comparison of dairy milk and PBAs is difficult because their prices typically do not reflect their environmental and nutritional health impacts, although PBAs tend to be significantly more expensive than dairy milk. Here, we integrate key results from the scientific literature using a True Cost Accounting (TCA) approach to compare dairy milk and five PBAs based on their market retail price and a quantification—and when possible, monetization—of key environmental, nutritional, and social impacts: Global Warming Potential (GWP), dietary risks, and forced labor, respectively. We compare whole dairy milk with five PBAs: soy, almond, oat, coconut, and pea, which account for 97% of retail market sales in the USA. The results show that while environmental, nutritional, and social benefits attributed to PBAs compared to dairy milk exist and can be significant, they are heterogenous, and for some PBAs, they may not be as significant as commonly perceived, particularly when the price premium they command are considered.

## 1. Introduction

Replacing animal-derived foods with plant-based foods is increasingly seen as an important step for achieving more sustainable and healthier food systems [1,2,3]. The substitution of dairy milk by plant-based alternative beverages (PBAs), promoted as alternatives to dairy milk, can be considered an important component of this process [2,4]. There is a growing market worldwide for PBAs, particularly in the USA and other developed countries [2,5,6]. These beverages, derived from a variety of plant species, are manufactured to replicate the sensory characteristics of dairy milk, such as a similar viscosity, white color, and creamy mouthfeel, while attempting to match important nutrient characteristics by fortifying them and enhancing the calcium and protein content to be similar to dairy milk [7,8]. The growth in consumption and sales of PBAs has happened even though they are substantially more expensive than dairy milk [2,6,9] and not nutritionally equivalent to it, for example, due to their lower protein content and quality, high carbohydrate and sugar content, limited nutrient content, even when fortified, as well as their content of phytic acid that can limit the bioavailability of essential minerals, even when present in the products, though all these characteristics vary among specific PBAs [10,11,12,13]. Although there are multiple motives for consuming PBAs, such as taste, digestibility, and lactose intolerance [14], the interest of consumers in sustainability and concerns for a better environment are important reasons for purchasing them [11]. Consumers of PBAs consider them better for the environment, human health, and animal welfare [8,11,15,16,17]. Consumers’ perceptions of PBAs as nutritionally and/or environmentally positive, however, may not be entirely supported by scientific evidence [2].

The recognition of the potential contribution of PBAs for making food systems more sustainable and healthier, as well as their increasing popularity among consumers, has led to a growing scientific literature comparing the environmental impacts and nutritional characteristics of dairy milk to different PBAs, as reviewed by Berardy et al. [7], Carlsson Kanyama et al. [18] and Silva and Smetana [19]. Most studies combine the results from Life Cycle Assessments (LCAs) for the environmental impacts with the nutritional composition reported in databases such as the USDA FoodData Central database (https://fdc.nal.usda.gov/ 17 January 2025), direct nutrient analyses, or product nutritional labels from dairy milk and different types of PBAs. The results from the LCAs vary substantially among studies, even for the same beverage, due to the use of different functional units, system boundaries, and impact assessment methodologies [7,18,19], complicating comparisons among studies and limiting the conclusions that can be drawn from them. The results from comparisons of nutritional composition show that dairy milk and PBAs differ significantly and are not nutritionally equivalent [10,13,20,21]. In addition to their environmental and nutritional differences, the higher prices of PBAs compared with dairy milk can be attributed to their higher marketing costs to attract consumers, higher investment in R&D, more expensive packaging, less streamlined logistics to process and distribute the product, as well as smaller market size and limited potential for economies of scale compared to the dairy industry [9]. Therefore, price differences reflect market conditions and do not necessarily capture differences in environmental and nutritional benefits and costs between dairy milk and PBAs, though these differences—particularly lower environmental impacts—are central for their marketing appeal to consumers [2,8,15,16]. Furthermore, for a consumer or a policy decisionmaker, it may be difficult to compare holistically the environmental impacts, nutritional health impacts, and market prices of dairy milk versus different PBAs and the tradeoffs involved, not only because of the different units used (e.g., kg CO_2_e, grams of polyunsaturated fats, etc.), but also because there are no markets associated with many of these impacts and therefore no market prices, i.e., they are “externalities” unaccounted for in the market. An externality is “a positive or negative consequence of an economic activity or transaction that affects other parties without being reflected in the price of the goods or services transacted” [22] (p. 29).

In this paper, we use a True Cost Accounting (TCA) approach to provide a more holistic comparison of dairy milk versus selected PBAs. TCA is an emerging economic assessment aimed at making visible, measuring, and—if possible—monetizing critical positive and negative externalities in food systems [23,24]. Externalities are assessed based on the reliance on, and effects of, an agri-food value chain in four capitals: natural, human, social, and produced [22]. Here, we integrate key results from the scientific literature, classifying them into these capitals and monetizing them, when possible, to quantify and examine the tradeoffs that a consumer may face when choosing between dairy milk and different PBAs. While the concept of monetization of externalities can be controversial [25,26], in TCA, the point of monetization is not to commoditize or privatize nature or other nonmarket aspects of food systems but to provide more transparency about the tradeoffs involved to make better and more sustainable decisions [27]. Monetization allows for the aggregation of very different impacts and a more transparent comparison among them [28]. This study focuses on whole dairy milk and on five PBAs with the following base ingredients: soy, almond, coconut, oat, and pea, in the context of the USA. These five PBAs were selected because they account for 97% of sales in the US market [29] and their data availability in the scientific literature.

## 2. Operationalizing Natural, Human, Productive, and Social Capitals

Natural capital refers to “the limited stocks of physical and biological resources found on earth, and of the limited capacity of ecosystems to provide ecosystem services” [22] (p. viii). Although natural capital refers to a wider set of issues, conventionally, its analysis has focused on environmental costs generated by a product or value chain due to the availability of data, particularly from LCAs [28]. As mentioned earlier, there are numerous studies comparing the environmental costs of PBAs and dairy milk using LCA methodologies, as reviewed by Berardy et al. [7], Carlsson Kanyama et al. [18] and Silva and Smetana [19]. There is variation in the types and magnitudes of environmental outcomes reported. Only coefficients for Global Warming Potential (GWP), which measures the impact of a product on climate change, were consistently reported across multiple studies.

Human capital refers to the knowledge, skills, competencies, and attributes embodied in individuals that facilitate the creation of personal, social, and economic well-being [22]. Human health is a central component of human capital because it is a prerequisite for the creation of personal, social, and economic well-being; a crucial aspect of human health is associated with diets and nutrition [28], which is particularly relevant in the case of PBAs and dairy milk. There is already a substantial literature examining nutritional differences between dairy milk and PBAs [7,10,11,13,20,21,30], which suggests that the effects of consuming PBAs versus dairy may lead to different nutritionally mediated health outcomes, though this will depend on the overall diet [2,31]. Therefore, here, we focus on comparing quantitatively the nutritional health impacts of dairy milk and PBAs and relate our findings to this literature. We use the Health Nutritional Index (HENI) method [32]. This method is based on studies of global burden of disease (GBD). Burdens of disease are the primary way to analyze impacts on human health that can be tied to nutrition outcomes [33].

Produced capital refers to “all manufactured capital, such as buildings, machinery, physical infrastructure (roads, water systems), as well as all financial capital and intellectual capital (technology, software, patents, brands, etc.)” [22] (p. viii). Measuring produced capital is the most straightforward, since there are market prices already associated with it. To examine the market retail prices of dairy milk versus PBAs, we use price data collected and reported by Raszap Skorbiansky, Saavoss, and Stewart [6] for dairy milk, soy milk, and almond milk, complemented with data retrieved from the websites of two major US food retailers for oat, pea, and coconut milk for the USA.

Social capital “encompasses networks, institutions, shared norms, values and understanding that facilitates cooperation among groups” [22] (p. viii). Decent working conditions are an indicator of social capital because they involve the existence and enforcement of social rules and expectations that lead to higher human well-being in agri-food supply chains. The opposite of decent work is forced labor, defined as “situations in which persons are coerced to work through the use of violence or intimidation, or by more subtle means such as accumulated debt, retention of identity papers, or threats of denunciation to immigration authorities” [34]. Therefore, the magnitude of forced labor risks in a food value chain can be seen as an indicator of social capital present in it. Absence or low levels of forced labor indicate high levels of social capital and high levels of forced labor indicate the opposite. Therefore, in this paper, we focus on forced labor involved in the value chains of PBAs and dairy milk as an indicator of social capital. Other indicators were not used because the data available is not specific and representative of PBAs, not even of the base ingredients.

**Figure 1:**
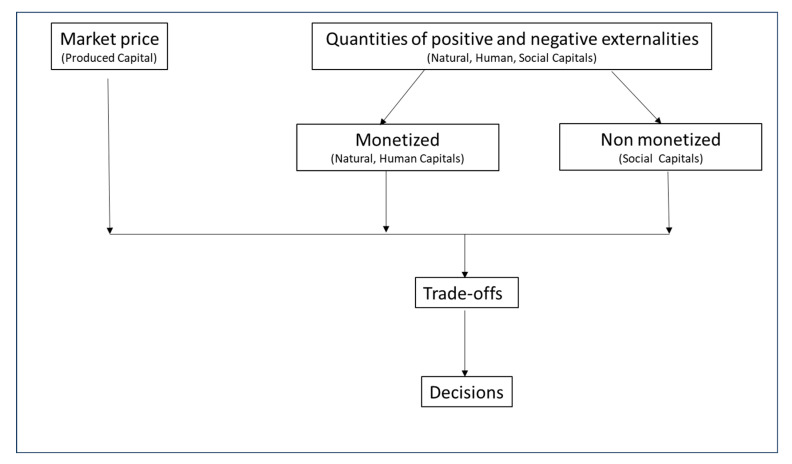
Framework for integrating impacts associated with the four capitals to analyze tradeoffs and reach decisions [28].

The results from GWP were monetized using the estimate of the social cost of CO_2_ reported by the US Environmental Protection Agency [35]. The results from the HENI were monetized using the monetization factor for a disability-adjusted life year (DALY) from True Price [36]. The results from forced labor cannot be monetized. We follow the same approach as in an earlier paper (Figure 1) [28].

## 3. Methods

### 3.1. Natural Capital: Global Warming Potential

From reviews of the literature comparing the environmental costs of PBAs and dairy milk [7,18,19], we identified 11 studies that compare dairy milk and five PBAs: soy, almond, oats, pea, and coconut, mostly focusing on the USA [3,37,38,39,40,41,42,43,44,45,46] (Supplementary Materials Table S1a). Most studies were LCAs of multiple beverages published as peer-reviewed journal articles but also included commissioned reports, class projects, and a thesis. As indicated above, there is variation in the types and magnitudes of environmental outcomes reported across studies. Only GWP coefficients are reported consistently (Supplementary Materials Table S1b) and are therefore the focus here. The units used in all studies were kg CO_2_ equivalents/functional unit (either liter or kg). Studies differed in system boundaries and functional units; therefore, for enhancing consistency and comparability among monetized GWP coefficients, we focus only on those with a cradle-to-retailer system boundary, which encompasses processes closest to the sale of the product and, thus, to market prices at retail. We converted all functional units to liters of beverage. We use an estimate of the social cost of greenhouse gases reported by the US Environmental Protection Agency [35] of USD_2020_ 0.120/kg CO_2_ for emissions that took place in 2020 as a monetization factor for GWP. For compatibility with other data in the paper, this factor was deflated to USD of 2022 using the Consumer Price Index for All Urban Consumers (CPI-U), not seasonally adjusted [47], resulting in a monetization factor of USD_2022_ 0.136/kg CO_2_.

### 3.2. Human Capital: Nutritional Health Impacts

To quantitatively compare the nutritional health impacts of dairy milk and PBAs, we relied on the HENI method [32]. This method is based on “the marginal health burden associated with 15 dietary risks, defined as dietary risk factors (DRFs) and expressed in disability-adjusted life years (DALYs) per g intake of risk component” (p. 616). DRFs were calculated for the diets of US adults (aged ≥25 years) [32]. It covers both health benefits as well as negative health impacts. We calculated the dietary risks associated with each beverage based on their content of the following nutrients: polyunsaturated fatty acids (PUFA), trans fatty acids (TFA), calcium, sodium, and fiber. In addition, Stylianou et al. (Supplementary Information of [32]) and [33] report a specific DRF for dairy milk, which we included in the calculations for dairy milk. The dairy milk DRF indicates a reduction in dietary risk due a net reduced risk of colorectal cancer and stroke while accounting for an increase in prostate cancer in males, associated with the consumption of dairy milk. We calculated the total dietary risk (TDR) of each beverage as follows (Equation (1)):

(1) $$TDRj=\sum_{i}DRFi\times Nij$$

where:

*TDRi* is the total dietary risk of beverage *j* in µDALY/gram.
*DRFi* is the dietary risk factor of nutrient *i* in µDALY/gram.
*Nij* is the content of nutrient *i* in beverage j in grams.

We obtained the content of these nutrients for dairy milk and the five PBAs from various sources (Supplemental Materials Table S2) and their corresponding DRFs from Stylianou et al. (Supplementary Information of [32]) (Supplemental Materials Table S3). The resulting TDR was converted to one liter (µDALY/liter). The TDR of each beverage reflects the avoided (benefit) or induced (cost) health burden associated with a marginal dietary change, e.g., the consumption of an additional reference amount of the beverage in the diet. It should be noted that dairy milk is the only beverage with trans fatty acids (TFA) among those studied here. Naturally-occurring TFA in milk (nTFA), however, are different from industrially produced TFA by the partial hydrogenation of vegetable oils (iTFA) [48,49,50]. While numerous harmful health effects have been documented as associated with the consumption of iTFA, nTFA have been shown to be less detrimental, and in some cases, even beneficial [48,49,50]. Furthermore, guidance on labelling from the US Food and Drug Administration (FDA) indicates that if the content of TFA is less than 0.5 g per serving, the content declared must be expressed as 0 g [51]. The TFA content of dairy milk is 0.279 g per serving [52], suggesting that according to the FDA criteria, TFA in dairy milk could be considered as “de minimis” (too small to be of significance). For these reasons, we calculated two TDRs for dairy milk: one including a dietary risk for TFA, as considered in the HENI method, and the other excluding it, assuming that their effect on health is neutral. Adding multiple dietary risks assumes “that the health effect from multiple dietary risks is independent and additive and that food components not covered by GBD have neutral health effects.” [32] (p. 617). Unlike the full HENI method, we did not rescale TDRs into minutes of healthy life gained or lost per reference amount of a beverage consumed. Instead, we multiplied the TDR per liter of a beverage by a monetization factor amounting to USD_2022_ 125,000/DALY [36]. According to the True Price Foundation, this factor is “based on a meta-analysis of the Value of Statistical Life (VSL) from 92 willingness-to-pay studies carried out by the OECD” [36] (p. 26) (see Supplementary Materials Text S1 for a description of the specific method and calculations).

### 3.3. Produced Capital: Retail Market Prices

Retail prices capture the value of a product at the consumer end of the value chain and implicitly are an indicator of the produced capital involved in its production. To examine the market retail prices of dairy milk versus PBAs, we use data collected and reported by Raszap Skorbiansky, Saavoss, and Stewart [6]. It is weekly store-based scanner data on prices of dairy, soy, and almond milk from 2013 to 2018 collected by IRI InfoScan. The sample covered on average over 60,000 stores per year. Weekly prices were averaged per year and deflated to USD of 2022 using the Consumer Price Index for All Urban Consumers (CPI-U), not seasonally adjusted [47]. We use the average retail deflated price for the period 2013–2018 per beverage for our analysis. Since no data was provided for oat, coconut, or pea milk, we gathered their prices as well as for dairy milk from the websites of two major US retailers and calculated the ratio of the price of each PBA to the price of dairy milk and then multiplied each ratio by the average retail price of dairy milk derived from Raszap Skorbiansky, Saavoss, and Stewart [6] data described above (see Supplementary Materials Text S2 for additional information).

### 3.4. Social Capital: Risk of Forced Labor

To assess the levels of forced labor involved in the value chains of PBAs and dairy milk in the USA, we use the approach and data from Blackstone et al. [53]. These authors have estimated the risk of forced labor for 212 food products—both locally produced and imported—consumed in the USA. The analysis is based on labor intensity, i.e., the number of working hours invested in producing one ton of a product in a particular country and a risk characterization factor of forced labor for that country. A quantitative characterization risk factor (CF) represents the relative probability of an adverse situation to occur [54], in this case forced labor. It is expressed relative to a medium risk level measured in “medium risk hours equivalents” (mrh-eq). We used the risk of forced labor as our indicator, calculated as follows Equation (2):

(2) RFLi=Li×CFi

where:

*RFLi* is the risk of forced labor of product *i* in mrh-eq/ton.
*Li* is the labor intensity of product *i* in hours/ton.
*CFi* is the characterization risk factor associated with product *i* in mrh-eq/hour.

For our analysis, we use the data from dairy whole milk and the base ingredients of the following PBAs: soy, almond, oat, coconut, and pea. We had to rely on the base ingredient of a PBA because there is no data for PBAs as such. Except for coconuts, which are imported from Mexico and Thailand, dairy milk and the other PBA-based ingredients were produced in the USA. A more detailed explanation of the methodology is presented in the Supplemental Material Text S3.

## 4. Results and Discussion

### 4.1. Natural Capital: Global Warming Potential

Table 1 presents the GWP, and associated monetized costs, of all beverages reported in the reviewed studies with a system boundary of cradle-to-retailer, as well as the averages per beverage (there is only one case each for coconut and pea milk). The results show that emissions—and their monetized values—from PBAs are a fraction of those of dairy milk. PBAs emissions are, on average, around 32.1% of dairy milk (varying between a minimum of 19.8% and a maximum of 58.6%).

### 4.2. Human Capital: Nutritional Health Impacts

**Figure 2:**
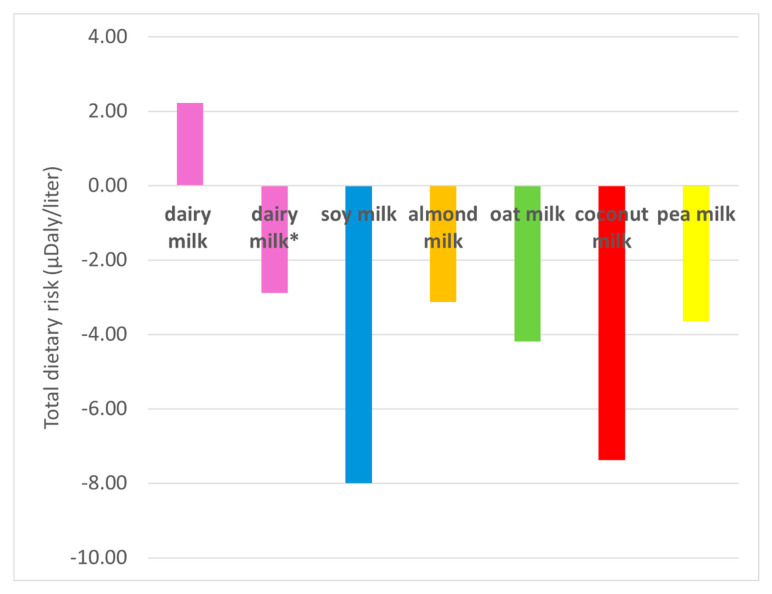
Total dietary risks of dairy milk and PBAs per liter. Negative numbers indicate a health benefit, while positive numbers indicate a health cost. Dairy milk* excludes TFA content from the calculations.

Figure 2 shows the TDRs of the different beverages based on their content of the relevant nutrients. In the case of TDRs, a negative number is a benefit (avoided health burden), and a positive one is a cost (induced health burden) associated with a marginal dietary change, e.g., the consumption of an additional reference amount of a product in the diet. All PBAs have negative TDRs, indicating a health benefit. The TDR for dairy milk depends on whether TFA are included or not in the calculation due to nutritional differences between natural TFA present in dairy milk from industrial TFA, as explained in the Methods section. If TFA are included, dairy milk’s TDR is positive, indicating a health cost, but if excluded, the TDR is negative, indicating a health benefit with a magnitude like almond milk (Table 2). The nutritional health benefits specific for dairy milk and PUFA content do not compensate for the costs associated with sodium content and of TFA if included. Soy milk shows the lowest dietary risk due to high PUFA and low sodium content. The nutritional health benefits of all other PBAs are associated with their high calcium content. These beverages are often fortified with this mineral [55]. However, while fortification may lead to similar or even higher levels of calcium of PBAs compared with dairy milk, calcium added through fortification of PBAs has lower bioavailability than naturally-occurring calcium in dairy milk [10,20]. The benefits from calcium in these beverages are reduced by their sodium content, with only almond and pea milk having benefits from PUFA content. The low total dietary risk of coconut milk compared to the other PBAs is related to its low sodium content.

Furthermore, the TDR calculations do not consider protein content or quality and other nutritional interactions that can be important for differences between dairy milk and PBAs. Dairy milk has the highest total protein content [10,21], with superior protein quality to all PBAs, though soy milk is close [10,13,30,56].

Monetization of the total risk factors shows a mirror image of Figure 2, since it is just scaling the total dietary risks by the monetization factor of a DALY and multiplied by −1 so that positive numbers indicate a health cost (the monetary value of an induced health burden), while negative numbers indicate a health benefit (the monetary value of the avoided health burden) (Supplemental Materials Figure S1).

### 4.3. Produced Capital: Retail Market Prices

**Figure 3:**
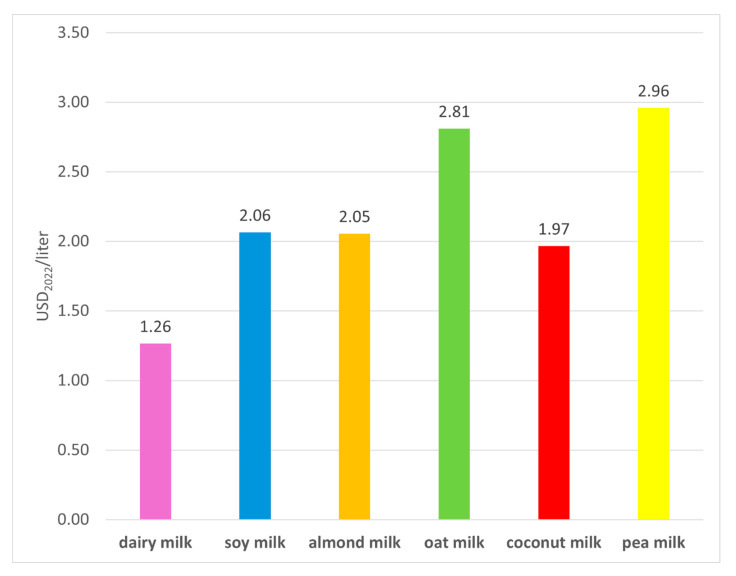
Average market retail prices for dairy milk and PBAs from 2013-2018 deflated to USD_2022._ Prices for oat, coconut, and pea milk were estimated from the ratios of observed retail prices of these beverages to dairy milk obtained from the websites of two major retailers in the USA. The number on top of the column is the market retail price of a beverage.

All PBAs have higher retail prices than dairy milk (Figure 3). Differences are on the order of 56% compared to coconut milk up to 135% compared to pea milk. Although the estimates for oat, coconut, and pea milk should be taken with caution since they assume that price ratios between these beverages and dairy milk have stayed constant between 2018 and 2024, they are based on observed ratios from retail prices retrieved from the websites of two major US retailers that show that these beverages are substantially more expensive than dairy milk (Supplemental Materials Figure S2).

### 4.4. Social Capital: Risk of Forced Labor

Table 3 shows the risk of forced labor and labor intensity for dairy milk and the PBAs’ base ingredients. Dairy milk and all base PBA ingredients are sourced in the USA, except for coconuts that are sourced in Thailand and Mexico, which account for 46.7% and 39.2%, respectively, i.e., 84.9%, of the total quantity imported into the USA. The results show that almonds have the highest risk of forced labor even if produced in the USA, about five times higher than dairy milk. All other PBAs base ingredients have lower risks of forced labor than dairy milk. The risk of forced labor for coconuts depends on the country of origin, with the lowest risk if sourced from Thailand but the third highest if sourced from Mexico. Soybeans, oats, and peas have a low risk of forced labor, probably because they are mechanized field crops with relatively low human labor intensity, thereby reducing the importance of social rules and their enforcement. However, for almonds, dairy milk, and coconuts, human labor intensity is higher and thus social rules and enforcement are more relevant. The case of coconuts is interesting since it suggests that social capital involved in their production is much higher in Thailand than in Mexico. It should also be noted that it is not possible to know for dairy milk if the risk of forced labor includes consideration of upstream production (e.g., cattle feed) or just of the dairy operation itself. Therefore, the risk of forced labor of dairy milk reported here may be conservative. This may be also the case for the PBAs, since their risk of forced labor is only based on that of their base ingredients and does not include their processing. These problems of underestimation are a limitation of this study.

### 4.5. True Cost and Tradeoffs

**Figure 4:**
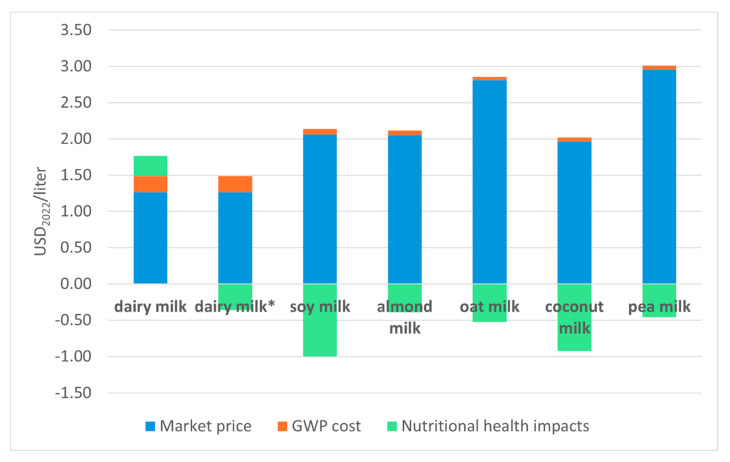
Market retail prices (blue), monetized costs of GWP (orange), and monetized nutritional health impacts (green) by beverage. A positive number indicates a monetary cost, while a negative number indicates a monetary benefit. Dairy milk* excludes TFA content from the HENI calculation of nutritional health impacts.

**Figure 5:**
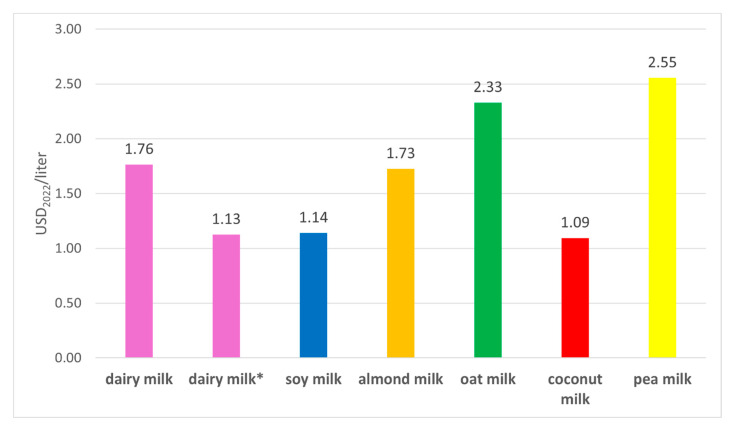
True cost of dairy milk and PBAs. The number on top of the column is the calculated true cost per liter. Dairy milk* excludes TFA content from the HENI calculation of nutritional health impacts.

Figure 4 shows the monetized cost of GWP and of the nutritional health impacts, as well as the retail market price for each of the beverages. The cost of GWP is the highest for dairy milk, while it is lower for the PBAs, as expected. Nutritional health impacts are beneficial for all beverages, except dairy milk if TFA are included. Therefore, in terms of the monetized externalities analyzed here, PBAs are superior to dairy milk. However, the market retail prices of PBAs are between 56 and 135% higher than dairy milk. Adding the monetized value of these externalities to the market retail prices of these beverages to reflect their true cost alters the assessment. For example, adding the cost of GWP to the market retail price of these beverages indicates that dairy milk has the lowest cost despite having a much higher cost of GWP. If nutritional health impacts are considered as well, pea and oat milk have the highest true cost (Figure 5). Dairy milk has the third highest true cost if TFA are included but the fifth if they are excluded, below almond and even soy milk. Coconut milk has the lowest cost (underlying data for both figures presented in Supplemental Materials Table S6). The results are similar using the retail prices collected from the websites of two major US retailers (Figures S3 and S4).

**Figure 6:**
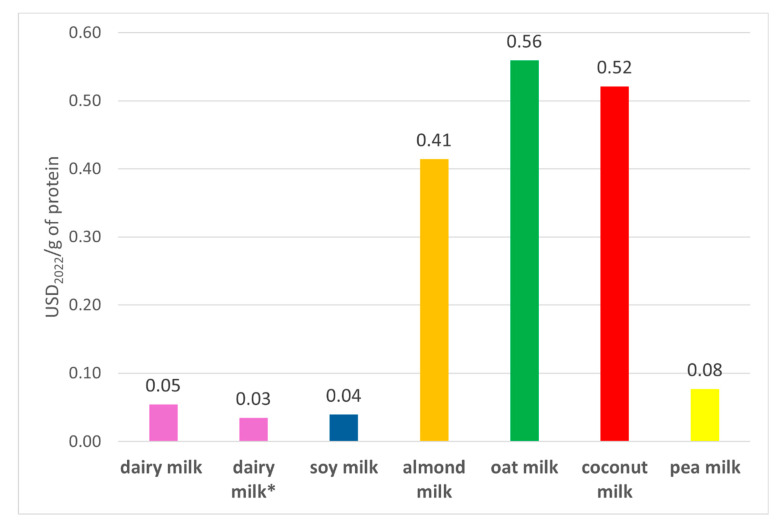
True cost per gram of protein of dairy milk and PBAs. The number on top of the column is the calculated true cost per gram. Dairy milk* excludes TFA content from the HENI calculation of nutritional health impacts.

The way nutritional health impacts were calculated does not consider protein content and quality. Dairy milk has a higher quantity of protein than all PBAs, except for pea milk, which is only marginally higher (Supplemental Materials Figure S5). Figure 6 shows the true cost per gram of protein by beverage. If TFA are included in the calculations for dietary risk of dairy milk, soy milk has the lowest true cost. However, if TFA are excluded, dairy milk has the lowest true cost. Almond, oat, and coconut milk have substantially higher true costs than dairy milk, soy milk, and pea milk. The high protein content of pea milk compensates for its high true cost. These comparisons do not consider differences in protein quality. Based on the Digestible Indispensable Amino Acid Score (DIAAS), a widely used metric to assess protein bioavailability [13], dairy milk has a score > 100% for all essential amino acids for children aged >3 years, adolescents, and adults and is considered excellent [13]. Soy milk also has a score > 100%, but oat (59.1%) and almond (39%) milk have much lower scores [13]. Although not specific for pea milk, pea protein can meet all amino acid requirements, but it has a lower score than casein, the main protein in dairy milk [56,58]. These results indicate that soy and dairy milk are the cheapest sources of protein, even accounting for the externalities examined here.

**Figure 7:**
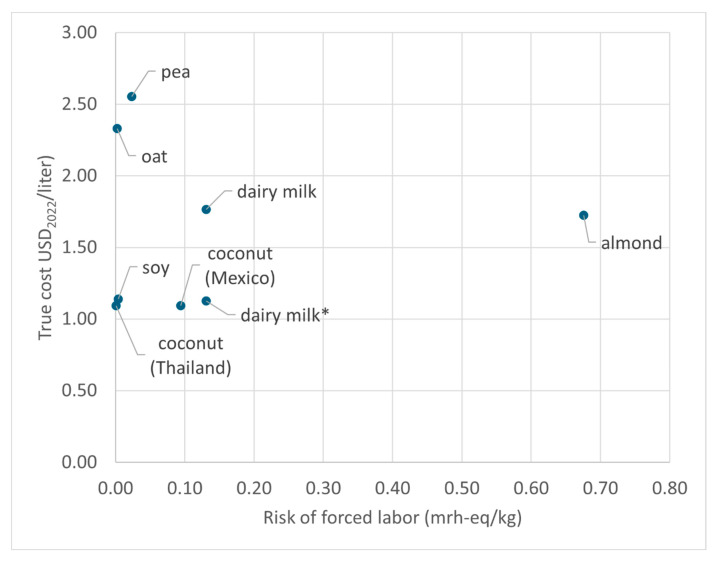
True costs of dairy milk and the PBAs compared to the levels of risk of forced labor of dairy milk and PBAs’ base ingredients. Dairy milk* excludes TFA content from the HENI calculation of nutritional health impacts.

Comparing the results from the true cost of these beverages with the risk of forced labor associated with their base ingredients (Figure 7) indicates that almond milk has a high true cost and the highest risk of forced labor. Compared with almond milk, dairy milk has a substantially lower risk of forced labor but a slightly higher true cost. The risk of forced labor for dairy milk refers to the finished product. In the case of PBAs, the risk of forced labor refers to their base ingredients, ignoring the processing phase of their production. The case of coconut milk indicates that social capital may be important and that there are substantial differences between the countries where the base ingredient is produced. In any case, and with the limitations of using forced labor as an indicator of social capital and the possible underestimation of the risks of forced labor noted above, our results suggest that levels of social capital should make a higher contribution to the true costs of dairy milk, almond, and coconut milk but less so to the other PBAs.

An issue that we did not incorporate in our analysis, and this is one of its limitations, was accounting for government subsidies that dairy milk production receives in the USA. The US Department of Agriculture (USDA) assists and promotes the US dairy industry through different programs that support dairy farmers to manage market risks and protect their revenue, donation programs, and various food purchase programs among others [59]. Of USD 161 billion provided in fiscal assistance to agricultural producers by the USDA for fiscal years 2019–2023, dairy producers received USD 7 billion (4.3%, with an average of USD 1.4 billion per year) [60]. Complicating matters is the fact that some of the crops that are the base ingredients for the PBAs may also benefit from some government support. Although the inclusion of government subsidies in the calculations of the true costs of dairy milk and PBAs may alter the assessment, addressing this issue is complex and beyond the scope of our study. This is an area that merits further consideration.

## 5. Conclusions

Our paper is based on the premise of True Cost Accounting, i.e., that our food systems generate important impacts on society that are unaccounted in market prices (externalities), leading to sub-optimal decisions by producers, consumers, and policy makers. We have examined this issue in the context of the externalities generated by an animal product (dairy milk) versus PBAs that aim to play similar roles for consumers and that are considered by many of them as generating less externalities than the animal product. We advance current knowledge by monetizing some of these externalities. While monetizing GWP is relatively straightforward, monetizing the nutritional health benefits and costs of these beverages is not. We applied the HENI method for this comparison, which, to our knowledge, has not carried out before in this context. We show in monetary terms how valuable PBAs are due to their nutritional benefits compared to dairy milk. While the HENI method relies on important assumptions and has limitations (see below), we also tried to provide a more complete perspective by presenting information on protein content and quality. We provide this information quantitatively by connecting protein content to the monetary true cost we calculated and qualitatively by referring to results from the literature. PBAs are more expensive than dairy milk, which is a cost that a large segment of consumers absorb as they consider the choice between dairy milk and PBAs. What consumer may not know is how much of a premium they pay for PBAs compared to the environmental and nutritional advantages that PBAs offer over dairy milk. We have aimed to present a liter-to-liter comparison to show what these premium prices deliver to consumers versus dairy milk. To the extent of our knowledge, we are the first ones to shed light on the magnitudes of some of these externalities in comparison to the premiums paid for PBAs relative to dairy milk. In addition, this is the first study to relate the production of PBAs and dairy milk to the forced labor involved in their production as an indicator of social capital. While we recognize that there are limitations in the method, our results provide additional information to compare these beverages.

Our findings indicate that the choice among dairy milk and PBAs is more complex and nuanced that it may seem at first sight when environmental, nutritional health, and social impacts—usually unaccounted for—are included in an assessment. Though there is a consensus that PBAs generate lower greenhouse gas emissions and therefore have a much lower impact on climate change than dairy milk—an important consideration for PBA consumers, a fact supported by our results—when quantified in monetary terms, these differences are substantially reduced by the premium prices they command. While there is a consensus that dairy milk is nutritionally superior to most PBAs, our results show that dairy milk can be associated with nutritional health costs. However, this depends on whether the calculations of TDRs include TFA or not, and there are reasons to exclude them. If TFA are excluded, dairy milk has nutritional health benefits like almond milk, though smaller than soy, oat, and pea milk.

Differences in health nutritional impacts among beverages, and therefore their comparative assessment, depend on the assumptions and limitations of the HENI method. This method is based on a narrow set of nutrients and assumes a linear association to health impacts, ignoring interactions among nutrients and potential nonlinear relationships between a nutrient and a health effect, as well as overlooking qualitative differences within a nutrient and assuming neutral health effects of all other nutrients not included in the calculation of TDR. In particular, qualitative variations within a nutrient and interactions among multiple nutrients can be substantially different between PBAs and dairy milk [10,13,20,21]. As indicated by Thorning et al. [21] (p. 6). “[T]he importance of studying whole foods instead of single nutrients is becoming clear as potential nutrient-nutrient interactions may affect the metabolic response to the whole food compared to its isolated nutrients.” For this reason, it is likely that our results underestimate the health nutritional benefits of dairy milk and their monetary value, as well as overestimate the health nutritional benefits of PBAs, for example, due to their content of phytic acid that limits the bioavailability of essential minerals [13]. In addition, a recent study by Ramsing et al. [11] has shown that dairy milk has a lower unit costs and higher nutrient value for protein and most micronutrients compared to various PBAs in the USA. In fact, we showed that dairy milk is one of the cheapest sources of protein even when accounting for the externalities examined here, as others have found using a different approach [30].

These issues suggest that methods based on the burdens of disease associated with differences in certain nutrients, such as the HENI method, may provide a limited comparative assessment of multiple foods. However, these methods provide a useful, if partial, approach to quantifying and monetizing nutritional health impacts. Further research is needed to resolve these diverging findings.

The true costs calculated here, however, are only approximations, since we estimated them using only three factors—GWP, dietary risk, and forced labor—while there are many others that were not considered here due to the availability of data. Despite this limitation, our results show that while environmental, nutritional, and social benefits attributed to PBAs compared to dairy milk exist and can be significant, they are heterogenous and for some PBAs they may not be as substantial as commonly perceived, particularly when the price premiums they command are considered.

## Figures and Tables

**Table 1:** GWP and associated monetized value of beverages reported by studies with a cradle-to-retailer system boundary.

Study	Beverage	GWP (kg CO_2_e/L)	Cost (USD_2022_/kg CO_2_e)
Poore and Nemecek [3]	dairy milk	3.20	0.43
Geburt et al. [40]	dairy milk	1.37	0.19
Geburt et al. [40] (organic milk)	dairy milk (organic)	1.41	0.19
Henderson and Unnasch [42]	dairy milk	1.47	0.20
Clune et al. [39]	dairy milk	1.13	0.15
Clune et al. [39] (organic dairy milk)	dairy milk (organic)	1.95	0.26
Smedman et al. [43]	dairy milk	0.96	0.13
Average	dairy milk	1.64	0.22
Poore and Nemecek [3]	soy milk	1.00	0.14
Geburt et al. [40]	soy milk	0.45	0.06
Henderson and Unnasch [42]	soy milk	0.40	0.05
Clune et al. [39]	soy milk	0.66	0.09
Smedman et al. [43]	soy milk	0.29	0.04
Average	soy milk	0.56	0.08
Geburt et al. [40]	almond milk	0.59	0.08
Henderson and Unnasch [42]	almond milk	0.37	0.05
Clune et al. [39]	almond milk	0.41	0.06
Average	almond milk	0.46	0.06
Geburt et al. [40]	oat milk	0.45	0.06
Smedman et al. [43]	oat milk	0.20	0.03
Average	oat milk	0.33	0.04
Clune et al. [39]	coconut milk	0.38	0.05
Henderson and Unnasch [42]	pea milk	0.39	0.05

**Table 2:** Dietary risks of dairy milk and PBAs by nutrient and total in µDALY per liter.

	PUFA	TFA	Calcium	Sodium	Fiber other	Milk	Total
Dairy milk	−0.67	5.11	0 ^2^	5.48	0	−7.70	2.22
Dairy milk*	−0.67	0 ^1^	0 ^2^	5.48	0	−7.70	2.89
Soy milk	−6.25	0	−6.38	4.63	−0.01	0	−8.00
Almond milk	−1.25	0	−9.99	8.11	0	0	−3.13
Oat milk	0.00	0	−9.99	5.79	0	0	−4.20
Pea milk	−1.25	0	−9.35	6.95	0	0	−3.65
Coconut milk	0.00	0	−9.99	2.61	0	0	−7.38

^1^ TFA excluded, assuming that their health effects are neutral. ^2^ The DRF for calcium is excluded in the calculations for dairy milk to avoid double counting the health benefits on colorectal cancer already captured in the specific DRF for dairy milk (Supplementary Information of [32]). Dairy milk* excludes TFA content from the calculations.

**Table 3:** Risk of forced labor of PBAs’ base ingredients and whole dairy milk.

**Product**	**Labor Intensity (Hours/kg)**	Risk of Forced Labor (Mrh-eq/kg)	Ratio of the Risk of Forced Labor of PBA to Dairy Milk
Dairy milk	0.013	0.131	1.000
Soybeans	0.001	0.004	0.030
Almonds	0.135	0.676	5.154
Oats	0.001	0.002	0.016
Coconuts (Mexico) ^1^	0.019	0.094	0.717
Coconuts (Thailand) ^2^	0.055	0.001	0.004
Peas	0.005	0.023	0.178

^1^ Labor intensity: the number of working hours invested to produce one kg of a product. ^2^ Thailand and Mexico were the highest suppliers of coconuts with 46.7% and 39.2%, respectively, accounting for 84.9% of the total reported for the USA. Source: [57], Supplementary Materials dataverse_files, SI_upstream_paths.csv. https://doi.org/10.7910/DVN/LEVNP2 (accessed 13 February 2025).

## Data Availability

The original contributions presented in the study are included in the article/Supplementary Materials, further inquiries can be directed to the corresponding author.

## Supplementary Materials

The following supporting information can be downloaded at: https://www.mdpi.com/article/10.3390/foods14132196/s1, Text S1. Methodology for calculating total dietary risks. Text S2. Additional information on retail prices. Text S3. Methodology for calculating forced labor. Table S1a,b. Reviewed studies on environmental impacts of PBAs and dairy milk. Table S2. Content of key nutrients for beverages analyzed. Table S3. Dietary risks factors. Table S4. Conversion to qualitative risk level to characterization risk factors. Table S5. Conversion factors used to convert known occurrences of forced labor events into qualitative risk levels. Table S6. Underlying data for Figure 3 and Figure 4. Figure S1. Monetization of dietary risks of dairy milk and PBAs per liter. Figure S2. Observed retail prices for the different beverages retrieved from the websites of two major US retailers. Figure S3. Retail prices, monetized costs of GWP, and monetized nutritional health impacts by beverage using retail prices retrieved from the websites of two major US retailers. Figure S4. True cost of dairy milk and the PBAs retrieved from the websites of two major US retailers. Figure S5. Grams of protein content per liter of beverage. References [61,62,63] are cited in the supplementary materials.
