# Supplementary material for: Towards a More Holistic Comparative Assessment of Plant-Based Alternative Beverages and Dairy Milk: A True Cost Accounting Approach
Source: Foods. 2025 Jun 23;14(13):2196. doi: 10.3390/foods14132196 (PMC12248856; doi:10.3390/foods14132196)
Supplement: Supplementary file 1 [file foods-14-02196-s001.zip › foods-3676634-supplementary.pdf]

Supplementary Materials for the paper "Towards a more holistic comparative assessment of Plant-based alternative beverages and dairy milk: A True Cost Accounting approach."

Table S1a. Reviewed studies on environmental impacts of PBAs and dairy milk

**Author**	**Country/region**	**Products compared**	**Functional unit**	**System boundary**
Poore and Nemecek [1]	World	Dairy Milk, Soy	1 liter of product	Cradle to grave
te Pas and Westbroek [2]	Utah, USA	Oat, Dairy Milk	1 liter of product	Cradle to grave
Geburt et al. [3]	Switzerland, USA, Brazil	Almond, Soy, Oat, Conventional & organic Dairy Milk	1 kg of product	Cradle to retailer
Henderson and Unnash [4]	USA	Almond, Pea, Soy, Dairy Milk	1 liter of product	Cradle to retailer
Ho et al. [5]	USA	Almond, Dairy Milk	1 liter of product	Cradle farm gate
Clune et al. [6]	USA	Almond, Coconut, Conventional & organic Dairy Milk, Soy	1 kg of product	Cradle to retailer

Chapa et al. [7]	USA	Dairy Milk, Soy	1 liter of product	Cradle to factory gate
Heller et al. [8]	USA	Dairy Milk, Soy	1 kg of product	Cradle to factory gate
Smedman et al. [9]	Sweden	Dairy Milk, Soy, Oat	1 kg of product	Cradle to retailer
Beckerman et al. [10]	USA	Dairy Milk, Soy	1 kg of product	Cradle to grave
Werner et al. [11]	Denmark	Dairy Milk, Soy	1 kg of product	Cradle to grave

Table S1b. Reviewed studies on environmental impacts of PBAs and dairy milk.

		Study										
**Environmental outcome**	**Units**	**[1]**	**[2]**	**[3]**	**[4]**	**[5]**	**[6]**	**[7]**	**[8]**	**[9]**	**[10]**	**[11]**
GWP	kg CO_2 e	✓	✓	✓	✓	✓	✓	✓	✓	✓	✓	✓
Water use (liter)	liter	✓	✓	✓	✓	✓						
Land use	m^2	✓	✓	✓								
Terrestrial acidification	kg SO_2 e		✓	✓								
Acidification potential (AP)	kg SO_2 e	✓										
Freshwater eutrophication	kg P e		✓	✓								
Marine eutrophication	kg N e		✓	✓								
Eutrophication potential (EP)	kg $PO4_3$ e	✓										
Fine particulate matter formation/ Human health particulate air (HHPA)	kg PM2.5 e		✓									
Ozone formation	kg NOx eq			✓								
Terrestrial ecotoxicity	kg 1,4-DCB			✓								
Freshwater ecotoxicity	kg 1,4-DCB			✓								
Marine ecotoxicity	kg 1,4-DCB			✓								

Mineral resource scarcity	kg Cu eq		√									
Fossil resource scarcity	kg oil eq		√									

Text S1. Methodology for calculating total dietary risks

To calculate the total dietary risks for dairy milk and the PBAs, the content of calcium, polyunsaturated fats (PUFA), trans-fatty acids (TFA), calcium, sodium and fiber per serving of 1 cup (240 ml) for each beverage was obtained from different sources (Table S2) and multiplied by their associated mean dietary risk factors (DRFs) (Table S3) extracted from Table S3 in the Supplementary Information of Stylianou et al. [12]. In the case of dairy milk, a specific DRF for milk, indicating that there is a reduction in dietary risk associated with dairy milk consumption [13]. The resulting factors were added to obtain a total dietary risk for each beverage (Table 2 main text). In this context, negative numbers indicate a benefit (µDALY avoided), and positive numbers indicate a cost (µDALY incurred).

Table S2. Content of key nutrients for beverages analyzed (all PBAs are unsweetened, except oat milk).

	Whole milk[1] (240 ml)	Soy[2] (240 ml)	Almond[3] (240 ml)	Oat[4] (240 ml)	Pea[5] (240 ml)	Coconut[6] (240ml)
PUFA g	0.269	2.5	0.5	0	0.5	0
TFA g	0.279	0	0	0	0	0
Calcium mg	306	300	470	470	440	470
Sodium mg	94.6	80	140	100	120	45
Fiber g	0	2	0	0	<1	0

Source: [1]https://fdc.nal.usda.gov/fdc-app.html#/food-details/746782/nutrients (accessed January 17, 2025). Nutritional label of: [2]Silk organic unsweet soymilk https://silk.com/plant-based-products/soymilk/organic-unsweet-soymilk (accessed January 17, 2025); [3]Silk unsweet almond milk https://silk.com/plant-based-products/almondmilk/unsweet-almondmilk/ (accessed January 17, 2025); [4]Silk original oatmilk (contains added sugars) https://silk.com/plant-based-products/oatmilk/oatmilk/ (accessed January 17, 2025); [5]Unsweetened original Ripple pea https://ripplefoods.com/products/ripple-shelf-stable-unsweetened-original-milk-1-pack (accessed January 17, 2025); [6]Silk unsweet coconut milk https://silk.com/plant-based-products/coconutmilk/unsweet-coconutmilk/(accessed January 17, 2025).

Table S3. Dietary risks factors (mean).

Dietary risk	DRF (μDALYs/g)
Polyunsaturated fatty acids (PUFA)	-0.6
Trans fatty acids (TFA)	4.4
Calcium	-5.1
Sodium	13.9
Fiber other	-0.99
Milk	-0.0077

Source: Table S3 in the Supplementary Information of Stylianou et al. [12].

Text S2. Additional information on retail prices

The data we used to calculate the retail prices of dairy milk and PBAs (2013-2018) [14] could be considered somehow dated. However, it was the only disaggregated data based on actual purchases that was available publicly and spanned multiple years. Therefore, the average prices we used are based on a relatively long-term series that considers price fluctuations during that period. We complemented this data with current price data directly collected from Walmart and Amazon websites for the dairy milk and the five PBAs, but only for one day. There is a high correlation between the data we calculated from Raszap Skorbiansky et al. [14] and the data we retrieved from the web for dairy milk, almond and oat milk (r=0.81). We also estimated the true cost of these beverages using the current data (discounted to USD_{2022} for comparability) and obtained very similar results (Figures S3 and S4).

Text S3. Methodology for calculating forced labor

Blackstone et al. [15,16] calculated the risk of forced labor by compiling data on labor intensity, i.e., the number of working hours invested to produce one ton of a product in a particular country that supplies the USA. Then they calculated a risk characterization factor (CF) for the product according to the country where it was sourced, whether it was produced or imported into the USA. The risk CF represents the relative probability of an adverse situation to occur [17] (p.19), in this case, forced labor, in the production of the product of interest. The relative probabilities are expressed in relations to the medium risk level as “medium risk hours equivalents” (for an explanation of the procedure to calculate the CF see below). Then the CF of a particular product and country is multiplied by the corresponding labor intensity (hours/ton). The resulting number is the unweighted risk of forced labor expressed in medium risk hours equivalents per ton (mrh-eq/ton). Then this number is multiplied by the fraction of the total amount of the product consumed in the USA to obtain the weighted risk of forced labor expressed in “medium risk hours equivalents” per ton (mrh-eq/ton). The weighted risk of forced labor for each of the countries where the product was produced is then summed up. This number is the total weighted risk of forced labor for the product. For processed products, similar calculations are done for each of the production stages involved in their production and then summed up.

The risk CF is calculated as follows [16]. From a review of relevant documents from organizations such as the International Labor Organization (ILO), the US State Department, Verité, Inc., as well as case studies, the academic literature, and compiling information from investigative journalism, the number of “known occurrences” of forced labor events in a country is recorded in three steps depending on the available information.

Step 1 refers to the known occurrences at the country-commodity level (e.g., avocados from Mexico). Step 2 refers to known occurrences at the sector-country level (e.g., fruit sector in Mexico). Step 3 refers to the country specific risk from the Global Slavery Index. In addition, a score on a government response to human trafficking is calculated from the government compliance with the United States’ Trafficking Victims Protection Act of 2000 (TVPA), as amended minimum standards for the elimination of trafficking. The compliance is reported by U.S. Department of State, Office to Monitor and Combat Trafficking in Persons [18]. This report

classifies governments into four tiers[1], a score is assigned to each of these four tiers. As an example, regarding avocados from Mexico, the country was classified as Tier 2 in the report of 2023 Department of State, Office to Monitor and Combat Trafficking in Persons [19].

The known occurrences from the highest resolution step of data available is selected and assigned a weight of 85%, while the score from a government response to human trafficking is assigned a weight of 15% [17] (p. 41). Both numbers are then added to obtain a quantitative weighted average. This weighted average score in turn is converted to a qualitative risk level of forced labor using the following cutoff points: > 3.0 very high, > 2.5 but ≤ 3.0 high, > 1.5 but ≤ 2.5 medium, > 1.0 but ≤ 1.5 low, and < 1.0 very low (these cutoff points are used by the Social Hotspots Database, [17] (p. 41)). Finally, the qualitative risk levels are converted to a quantitative characterization risk factor (CF). CF represents the relative probability of an adverse situation to occur, in this case forced labor [17] (p.19). The qualitative risk levels of forced labor are converted into CF as follows (Table S4). Relative probabilities are expressed in relations to the medium risk level as "medium risk hours equivalents."

---

[1] Tier 1: Countries whose governments fully meet the TVPA's minimum standards for the elimination of trafficking. Tier 2: Countries whose governments do not fully meet the TVPA's minimum standards but are making significant efforts to bring themselves into compliance with those standards. Tier 2 Watch list: similar to Tier 2 but the estimated number of victims of severe forms of trafficking is very significant or is significantly increasing and the country is not taking proportional concrete actions, or there is a failure to provide evidence of increasing efforts to combat severe forms of trafficking in persons from the previous year, including increased investigations, prosecutions, and convictions of trafficking crimes, increased assistance to victims, and decreasing evidence of complicity in severe forms of trafficking by government officials. Tier 3: Countries whose governments do not fully meet the TVPA's minimum standards and are not making significant efforts to do so. U.S. Department of State, Office to Monitor and Combat Trafficking in Persons [19] (p. 69).

Table S4. Conversion to qualitative risk level to characterization risk factor.

Risk level	Characterization risk (relative probability)
Very high	10
High	5
**Medium**	**1**
Low	.01

Source: Table 2 in [17] (p. 19).

Table S5 presents the scores used to convert known occurrences and government response to human trafficking into qualitative risk levels by Blackstone et al.[15]. Note that since the relative probabilities are expressed in relation to the medium risk level—which is equal to one by definition, this assumes that the baseline is the medium risk of an adverse situation to occur, i.e., forced labor. This in turn means that a very high risk is ten times the relative probability of a medium risk, while a high risk is five times.

Table S5. Conversion factors used to convert known occurrences of forced labor events into qualitative risk levels.

	Steps 1 and 2	Step 3 (% people enslaved)	Governance
	Known occurrences (85%)[a]		(15%)[a]
Very High	4		Tier 3
High	3	> 0.70	Tier 2
Medium	2	>0.30	Tier 2W

Low	1	>0.20	Tier 1
Very Low	0.5	<0.19	

Source: Table 1 in [15] (p. 597).

[a] weight given to the score. If the weighted average is >3.0 then Very High, if >2.5 then High, if >1.5 then Medium, if > 1.0 then Low, if <= 1.0 then Very Low.

To calculate the risk of forced labor for dairy milk and the base ingredients of the PBAs, we downloaded the data from the webpage Supplementary Information for Force labor risk is pervasive in the US land-based food supply (https://doi.org/10.7910/DVN/LEVNP2, accessed February 13, 2025), specifically from the spreadsheet "SI_upstream_paths.csv." From the spreadsheet, the following final products were selected: (1) Milk, whole fresh cow, (2) soya sauce, (3) almonds shelled, (4) oats rolled, (5) coconut, and (6) peas dry. Milk, peas, and coconut are the final products. While soybeans, almonds with shells, and oats are intermediate products used in the production of soy sauce, almonds shelled, and oats rolled, respectively. We selected only products produced in the USA, except for coconuts that were imported from Mexico and Thailand. For each product we obtained the following parameters: (1) labor intensity in hours per ton, and (2) characterization risk in mrh-eq/ hour, i.e., the risk CF. For final products, these parameters were final. However, since intermediate products were sent to other countries to produce the final products (that were then imported into the USA), there could be multiple parameters depending on where the intermediate product was sent to. For example, soybeans produced in the USA were exported to 8 countries to produce soy sauce that then was imported into the USA. So, there were specific labor intensity and risk CFs for each country where the soybeans were exported to. For our calculations, we averaged these parameters to obtain a single labor intensity and risk CF for soybeans produced in the USA. The same procedure was carried out for peas, and oats.

It should be noted that the variable risk of forced labor for product i (RFL_i), corresponds to the category "unweighted risk" of a product in the original method of Blackstone et al. [15], as explained in the first part of this section. The "unweighted risk" of a product is an intermediate

stage in the calculation before its risk is weighted by the quantity of the product consumed in the USA. However, because some of the base ingredients are intermediate products for other products that are of no interest to our study (e.g. soy sauce), for consistency across products we use the unweighted risk which we simply call risk of forced labor.

Table S6. Underlying data for Figures 3 & 4.

Beverage	Market retail price	GWP cost	Net nutritional impacts	True cost
dairy milk	1.26	0.22	0.28	1.76
dairy milk*	1.26	0.22	-0.36	1.13
soy	2.06	0.08	-1.00	1.14
almond	2.05	0.06	-0.39	1.73
oat	2.81	0.04	-0.52	2.33
coconut	1.97	0.05	-0.92	1.09
pea	2.96	0.05	-0.46	2.55

Dairy milk* excludes TFA content from the calculations.

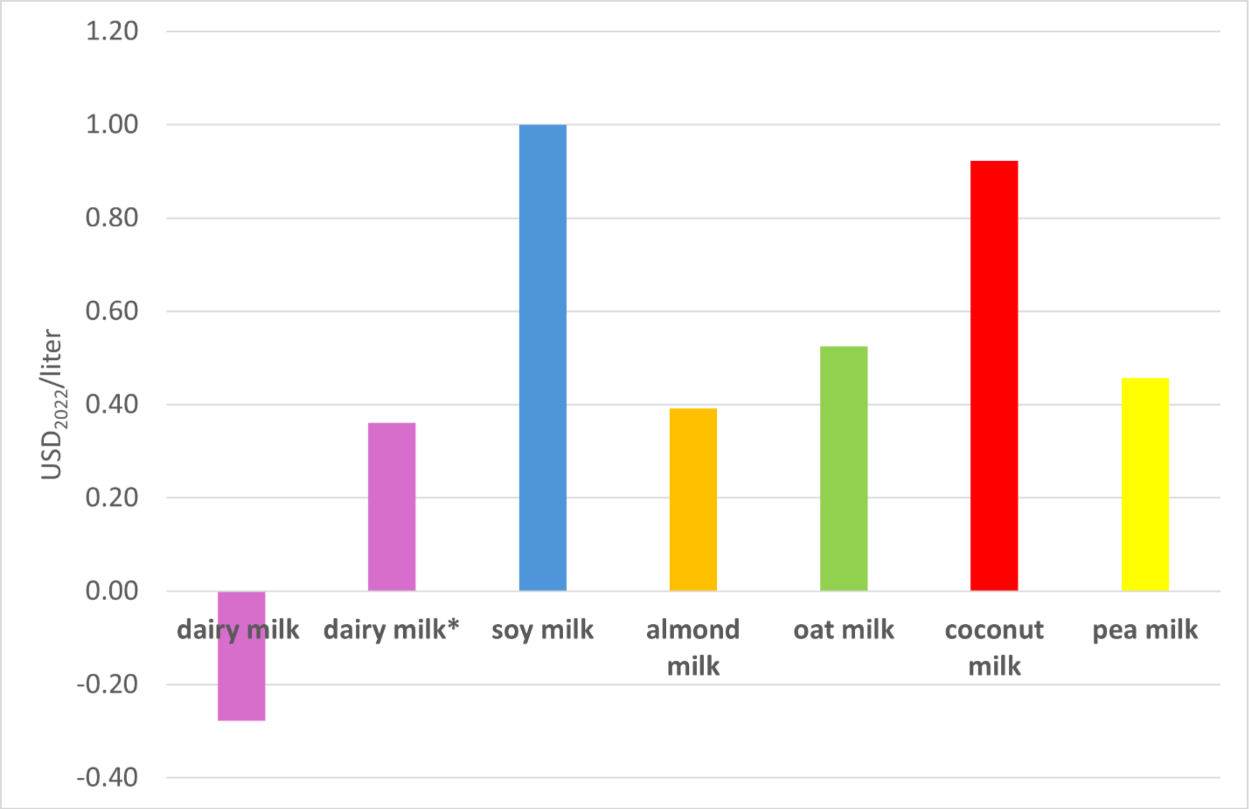

Figure S1. Monetization of dietary risks of dairy milk and PBAs per liter.

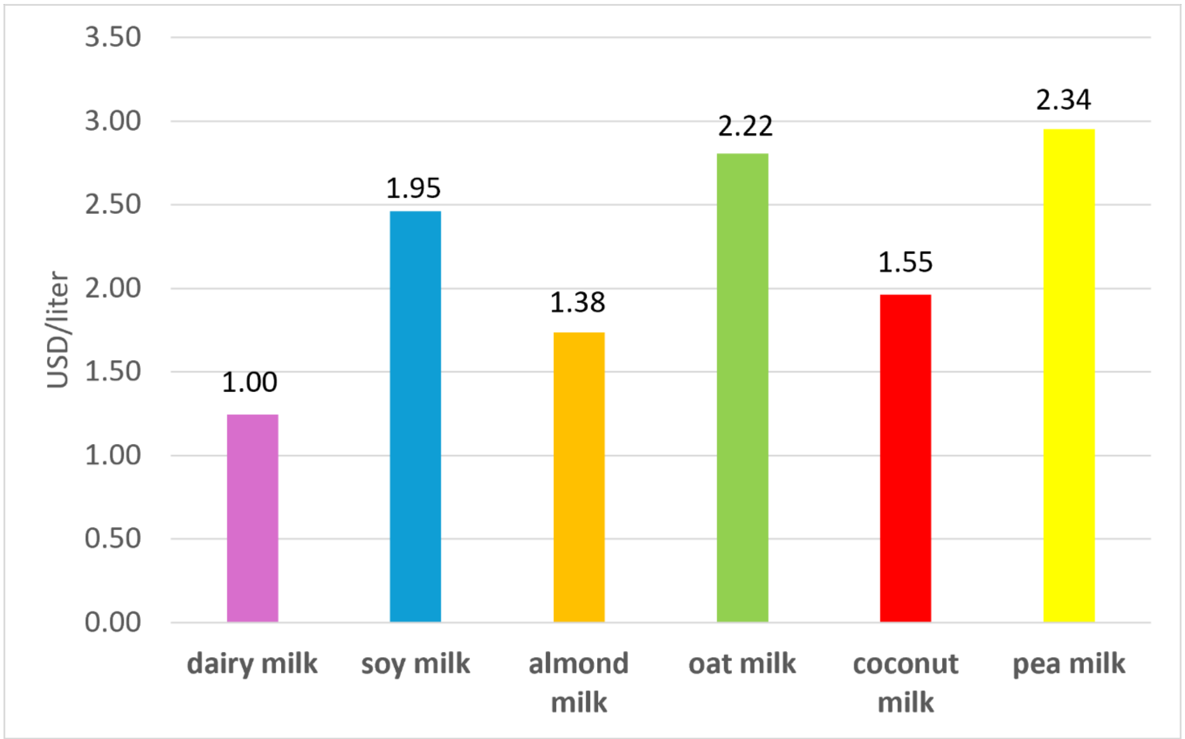

Figure S2. Observed retail prices for the different beverages retrieved from the websites of two major US retailers (Amazon and Walmart) on January 17, 2025, converted to USD/liter. The number on top of the column is the ratio of the price of the beverage to dairy milk. Sources: Whole milk Vitamin D half gallon, Shamrock (Walmart); Silk Soymilk Unsweetened Organic (64 oz.) Half Gallon (Walmart); Silk Almond Unsweetened (64 oz.) Half Gallon (Walmart); Silk Original Oatmilk (64 oz.) Half Gallon (Amazon; Silk Dairy Free, Gluten Free, Unsweet Coconut Milk, 64 fl oz Half Gallon (Walmart); Ripple, Pea Milk, Original, Unsweetened, 48 Oz (Amazon).

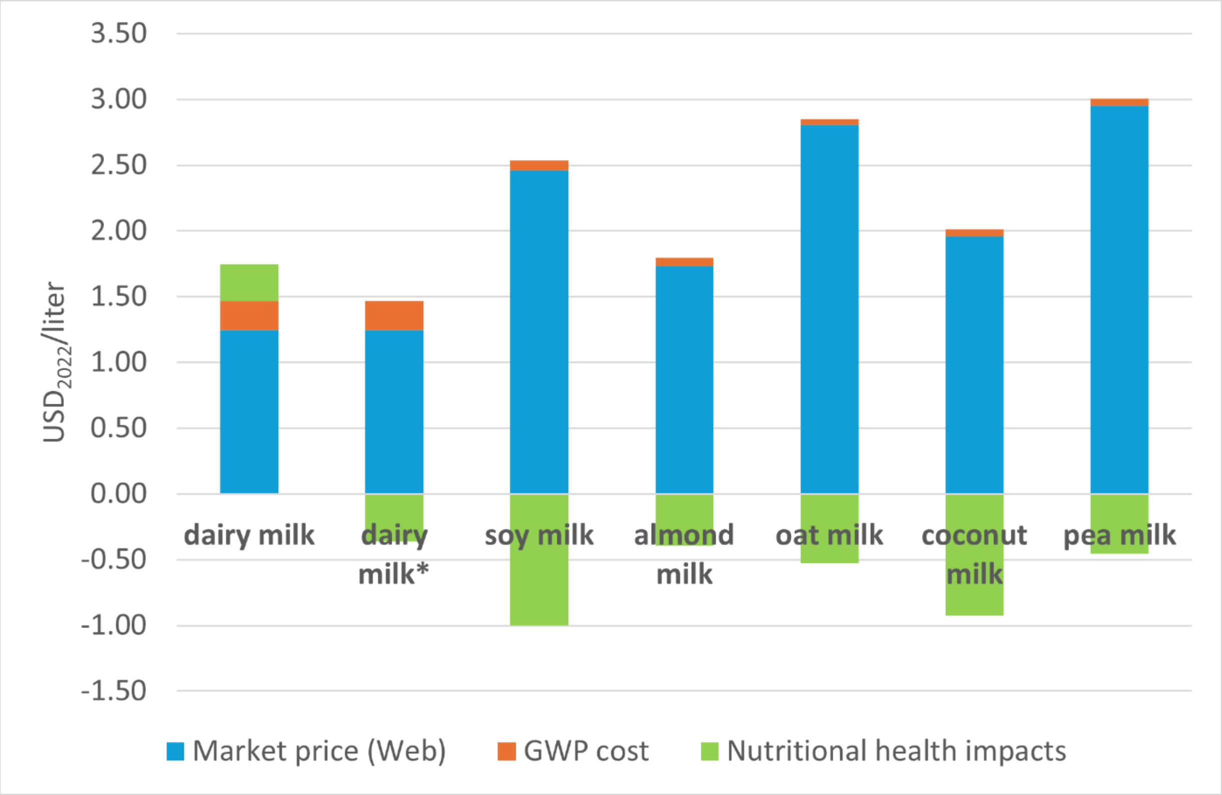

Figure S3. Retail prices, monetized costs of GWP, and monetized nutritional health impacts by beverage. Retail prices were collected from the websites of two major US retailers (Amazon and Walmart) on January 17, 2025, discounted to USD_{2022} and converted to USD/liter.

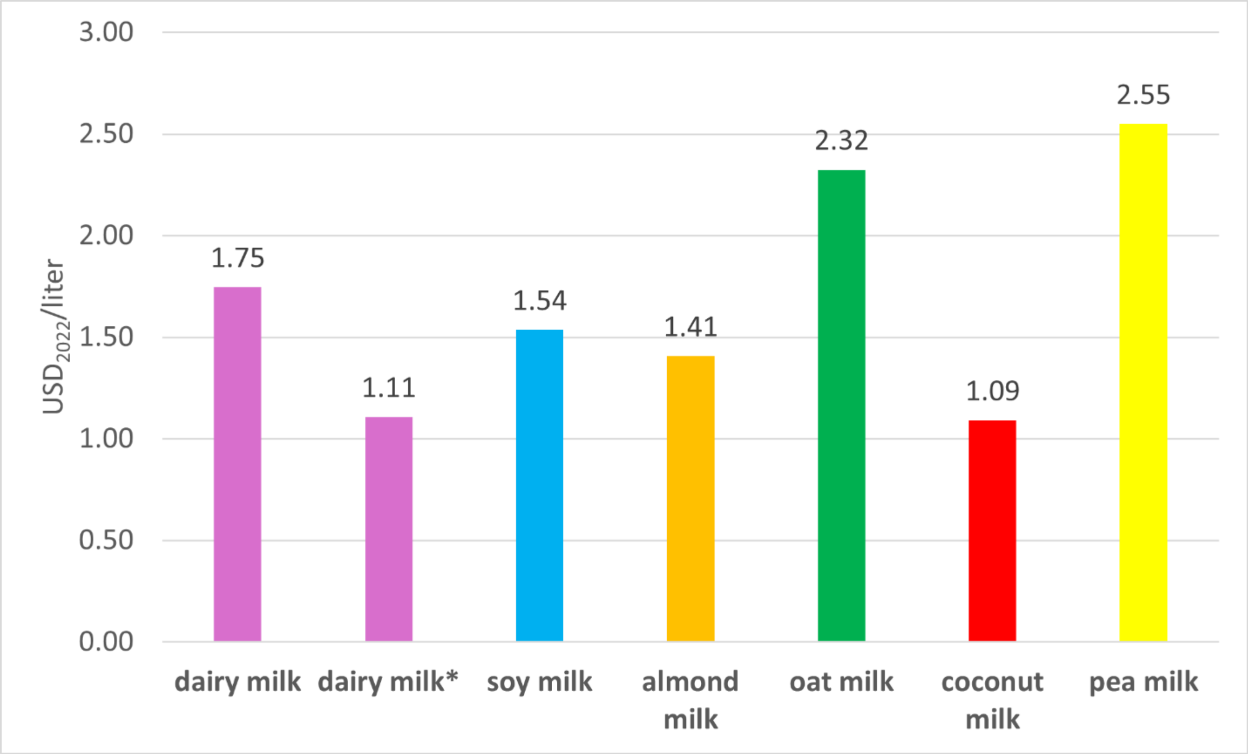

Figure S4. True cost of dairy milk and PBAs. The number on top of the column is the calculated true cost per liter. Retail prices were collected from the websites of two major US retailers (Amazon and Walmart) on January 17, 2025, discounted to USD_{2022} and converted to USD/liter.

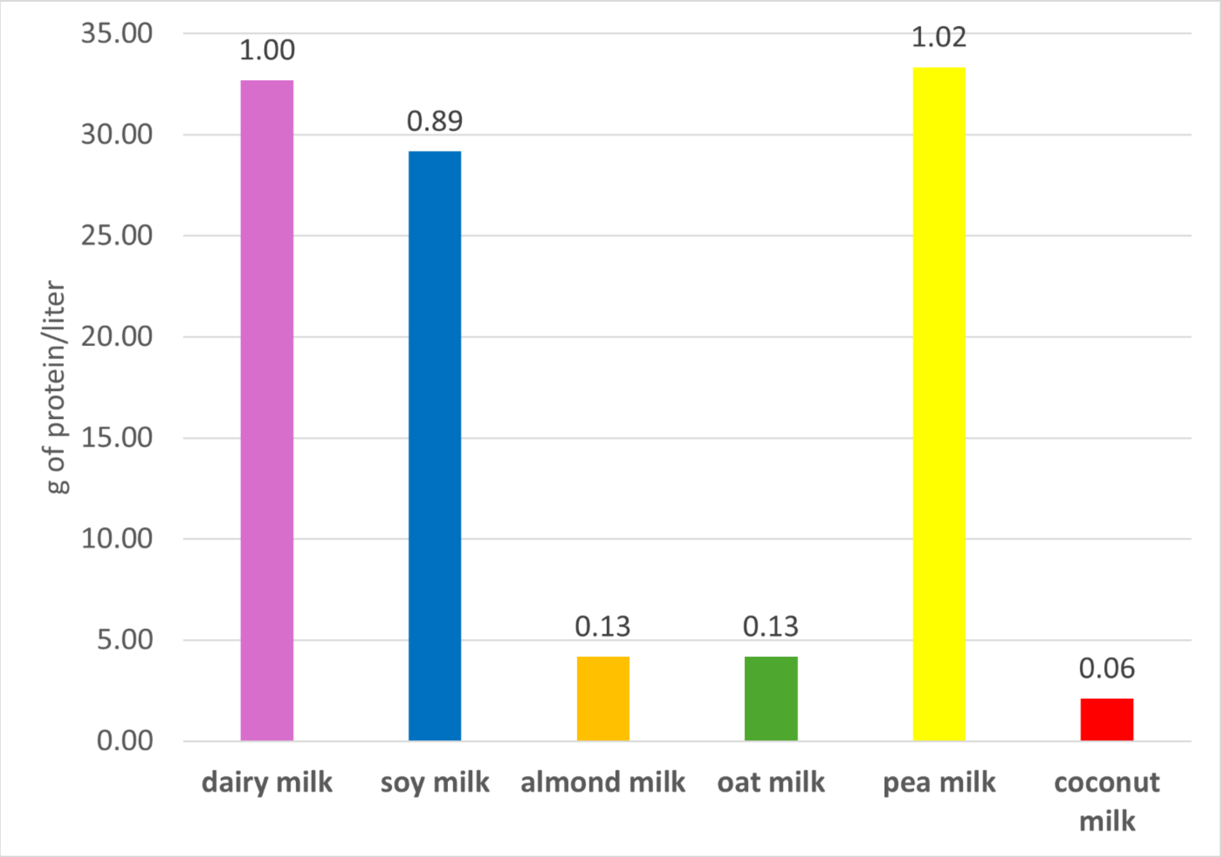

Figure S5. Grams of protein content per liter of beverage. The number on top of column is the ratio of protein content of a PBA/dairy milk.

Sources: dairy milk, https://fdc.nal.usda.gov/fdc-app.html#/food-details/746782/nutrients (accessed January 17, 2025); soy milk, https://fdc.nal.usda.gov/fdc-app.html#/food-details/2257044/nutrients (accessed January 17, 2025); almond milk, https://fdc.nal.usda.gov/fdc-app.html#/food-details/2257045/nutrients (accessed January 17, 2025); oat milk, https://fdc.nal.usda.gov/fdc-app.html#/food-details/2257046/nutrients; (accessed January 17, 2025); pea milk, https://www.ripplefoods.com/original-unsweetened-plant-milk/ (accessed January 17, 2025); coconut milk https://fdc.nal.usda.gov/food-details/174116/nutrients (accessed January 17, 2025; refers to coconut beverage sweetened, fortified with calcium, vitamins A, B12, D2, though in the rest of the paper we use unsweetened coconut milk, but it is likely that this does not make any difference in the protein content).

References

1. Poore, J.; Nemecek, T. Reducing Food’s Environmental Impacts through Producers and Consumers. *Science* **2018**, *360*, 987–992, doi:10.1126/science.aaq0216.
2. te Pas, C.; Westbroek, C. LCA of Oatly Barista and Comparison with Cow’s Milk 2022.
3. Geburt, K.; Albrecht, E.H.; Pointke, M.; Pawelzik, E.; Gerken, M.; Traulsen, I. A Comparative Analysis of Plant-Based Milk Alternatives Part 2: Environmental Impacts. *Sustainability* **2022**, *14*, 8424, doi:10.3390/su14148424.
4. Henderson, A.; Unnash, S. Life Cycle Assessment of Ripple Non-Dairy Milk 2017.
5. Ho, J.; Madariaga, I.; Martin, J.; Nguyen, H.; Trinh, L. UCLA IOES - Almond Milk vs. Cow Milk Life Cycle Assessment 2016. https://www.scribd.com/document/424896771/UCLA-IOES-Almond-Milk-vs-Cow-Milk-Life-Cycle-Assessment-2016# (accessed on February 4, 2025).
6. Clune, S.; Crossin, E.; Verghese, K. Systematic Review of Greenhouse Gas Emissions for Different Fresh Food Categories. *Journal of Cleaner Production* **2017**, *140*, 766–783, doi:10.1016/j.jclepro.2016.04.082.
7. Chapa, J.; Farkas, B.; Bailey, R.L.; Huang, J.-Y. Evaluation of Environmental Performance of Dietary Patterns in the United States Considering Food Nutrition and Satiety. *Science of The Total Environment* **2020**, *722*, 137672, doi:10.1016/j.scitotenv.2020.137672.
8. Heller, M.C.; Willits-Smith, A.; Meyer, R.; Keoleian, G.A.; Rose, D. Greenhouse Gas Emissions and Energy Use Associated with Production of Individual Self-Selected US Diets. *Environ. Res. Lett.* **2018**, *13*, 044004, doi:10.1088/1748-9326/aab0ac.
9. Smedman, A.; Lindmark-Månsson, H.; Drewnowski, A.; Edman, A.-K.M. Nutrient Density of Beverages in Relation to Climate Impact. *Food & Nutrition Research* **2010**, *54*, 5170, doi:10.3402/fnr.v54i0.5170.
10. Beckerman, J.P.; Blondin, S.A.; Richardson, S.A.; Rimm, E.B. Environmental and Economic Effects of Changing to Shelf-Stable Dairy or Soy Milk for the Breakfast in the Classroom Program. *Am J Public Health* **2019**, *109*, 736–738, doi:10.2105/AJPH.2019.304956.
11. Werner, L.B.; Flysjö, A.; Tholstrup, T. Greenhouse Gas Emissions of Realistic Dietary Choices in Denmark: The Carbon Footprint and Nutritional Value of Dairy Products. *Food & Nutrition Research* **2014**, *58*, 20687, doi:10.3402/fnr.v58.20687.
12. Stylianou, K.S.; Fulgoni, V.L.; Jolliet, O. Small Targeted Dietary Changes Can Yield Substantial Gains for Human Health and the Environment. *Nat Food* **2021**, *2*, 616–627, doi:10.1038/s43016-021-00343-4.
13. Stylianou, K.S.; Heller, M.C.; Fulgoni, V.L.; Ernstoff, A.S.; Keoleian, G.A.; Jolliet, O. A Life Cycle Assessment Framework Combining Nutritional and Environmental Health Impacts of Diet: A Case Study on Milk. *Int J Life Cycle Assess* **2016**, *21*, 734–746, doi:10.1007/s11367-015-0961-0.
14. Raszap Skorbiansky, S.; Saavoss, M.; Stewart, H. Cow’s Milk Still Leads in the United States: The Case of Cow’s, Almond, and Soy Milk. *Agricultural Economics* **2022**, *53*, 204–214, doi:10.1111/agec.12700.
15. Blackstone, N.T.; Rodríguez-Huerta, E.; Battaglia, K.; Jackson, B.; Jackson, E.; Benoit Norris, C.; Decker Sparks, J.L. Forced Labour Risk Is Pervasive in the US Land-Based Food Supply. *Nat Food* **2023**, *4*, 596–606, doi:10.1038/s43016-023-00794-x.

16. Blackstone, N.T.; Norris, C.B.; Robbins, T.; Jackson, B.; Decker Sparks, J.L. Risk of Forced Labour Embedded in the US Fruit and Vegetable Supply. *Nat Food* **2021**, *2*, 692–699, doi:10.1038/s43016-021-00339-0.
17. Benoît Norris, C.; Bennema, M.; Norris, Gregory. The Social Hotspots Database: Supporting Documentation (Update 2019) 2019. https://www.google.com.hk/url?sa=t&source=web&rct=j&opi=89978449&url=https://nexus.openlca.org/ws/files/23286&ved=2ahUKEwiAyKCB7fKNAxW-dfUHHSH7ATUQFnoECBcQAQ&usg=AOvVaw2mUwwksJ-UnwMBrE4ux8MY (accessed on February 13, 2025).
18. U.S. Department of State, Office to Monitor and Combat Trafficking in Persons Trafficking in Persons Report, June 2019. Available online: https://www.state.gov/wp-content/uploads/2019/06/2019-Trafficking-in-Persons-Report.pdf (accessed on February 13, 2025).
19. U.S. Department of State, Office to Monitor and Combat Trafficking in Persons Trafficking in Persons Report, June 2023. Available online: https://www.state.gov/wp-content/uploads/2023/06/Trafficking-in-Persons-Report-2023.pdf (accessed on February 13, 2025).
